# Maintaining higher leaf photosynthesis after heading stage could promote biomass accumulation in rice

**DOI:** 10.1038/s41598-021-86983-9

**Published:** 2021-04-07

**Authors:** Sotaro Honda, Satoshi Ohkubo, Nan Su San, Anothai Nakkasame, Kazuki Tomisawa, Keisuke Katsura, Taiichiro Ookawa, Atsushi J. Nagano, Shunsuke Adachi

**Affiliations:** 1 Graduate School of Agriculture, Tokyo University of Agriculture and Technology, 3-5-8 Saiwaicho, Fuchu, Tokyo 183-8509 Japan; 2 Faculty of Agriculture, Ryukoku University, Yokotani 1-5, Seta Oe-cho, Otsu, Shiga 520-2194 Japan; 3 College of Agriculture, Ibaraki University, 3-21-1 Chuo, Ami, Inashiki, Ibaraki 300-0393 Japan

**Keywords:** Plant sciences, Natural variation in plants, Photosynthesis

## Abstract

Leaf photosynthetic rate changes across the growing season as crop plants age. Most studies of leaf photosynthesis focus on a specific growth stage, leaving the question of which pattern of photosynthetic dynamics maximizes crop productivity unanswered. Here we obtained high-frequency data of canopy leaf CO_2_ assimilation rate (*A*) of two elite rice (*Oryza sativa*) cultivars and 76 inbred lines across the whole growing season. The integrated *A* value after heading was positively associated with crop growth rate (CGR) from heading to harvest, but that before heading was not. A curve-smoothing analysis of *A* after heading showed that accumulated *A* at > 80% of its maximum (*A*_80_) was positively correlated with CGR in analyses of all lines mixed and of lines grouped by genetic background, while the maximum *A* and accumulated *A* at ≤ 80% were less strongly correlated with CGR. We also found a genomic region (~ 12.2 Mb) that may enhance both *A*_80_ and aboveground biomass at harvest. We propose that maintaining a high *A* after heading, rather than having high maximum *A*, is a potential target for enhancing rice biomass accumulation.

## Introduction

Rice (*Oryza sativa*) is one of the most important cereal crops worldwide. To meet the increasing demand for grain as the world’s population increases, rice productivity must be increased by ~ 50% relative to the current level by 2050^1,2^. The rice yield increases during the “green revolution” depended largely on the development of semi-dwarf cultivars with greater harvest index and on greatly increased N fertilizer application^3,4^. This strategy is reaching its limits, however, because harvest index is reaching its theoretical maximum and excess application of N fertilizer causes environmental pollution^5–7^. Further enhancement of grain yield must be achieved through increases of total biomass accumulation via improved radiation use efficiency without increased nutrient inputs^8^. Single-leaf photosynthesis has long been considered a target trait for increasing radiation use efficiency^6,9,10^. Recent studies have shown the importance of enhancing single-leaf photosynthesis and crop productivity in the field^11^; for example, the promoted recovery from photoprotection increased biomass production in tobacco (*Nicotiana tabacum*)^12^, and overproduction of ribulose-1*,*5-bisphosphate carboxylase/oxygenase (Rubisco) increased grain yield in rice^13^.

Using natural genetic resources could be a useful approach for improving photosynthesis^14–16^. Wide intraspecific variation in net CO_2_ assimilation rate per leaf area (*A*) has been found in several crop species, including rice^17–19^ and wheat (*Triticum aestivum*)^20,21^. The underlying genetic variations can be used in quantitative genetic analyses to identify genomic regions relating to leaf photosynthesis, facilitating DNA marker-assisted selection^14,16^. An important question in such an approach is whether the enhanced *A* effectively increases total biomass production and grain yield^22^. Positive close correlations of *A* with plant (or crop) growth rate, biomass production, and final yield through large-scale surveys of diverse sets of accessions have been reported in rice^19,23,24^, wheat^25,26^ and soybean (*Glycine max*)^27^. Simulation analyses showed that a 25% increase in single-leaf photosynthesis based on rice genetic resources could enhance biomass production by 22–29%^28^. Furthermore, newer rice cultivars developed in Japan with high yield capacity have higher *A* than older cultivars, especially after heading^29,30^. These studies underpin the potential for enhanced productivity by improved photosynthesis achieved through the use of natural genetic resources.

In contrast, there are many conflicting results on the photosynthesis–productivity relationship. Poor correlations between *A* and biomass accumulation have been reported in rice^18,31^, wheat^21^ and maize (*Zea mays*)^32^. For example, Jahn et al. found a significant negative correlation between *A* and dry biomass among 20 diverse rice varieties^18^. Our previous research also showed that a near isogenic rice line with enhanced *A* significantly reduced the grain yield than its parental cultivars^33^. Many agronomists have been questioned the effects of the genetic improvement of single-leaf photosynthesis for better crop yields^34,35^. In fact, crop breeding has often selected increased leaf area production at the expense of photosynthetic capacity, as occurred in wheat^36^. The inconsistencies between studies could reduce the potential value of natural genetic resources for improving leaf photosynthesis and delay the enhancement of crop productivity.

The value of *A* changes across the growing season owing to the progression of plant age and leaf senescence^37–39^. However, most studies of the photosynthesis–productivity relationship selected only one or two growth stages for evaluation of photosynthesis^18,19,26,30^. Such a “snapshot” analysis can reveal only limited aspects of crop production and potentially cause inconsistent results. The need for comprehensive evaluation is supported by the fact that the total CO_2_ uptake per tobacco plant, calculated from multiple measurements of leaves at several positions throughout the day and the growing season, agreed well with actual dry weight increase^40^. Therefore, multiple photosynthetic measurements are necessary when we examine natural genetic resources across their growing season.

Conventional open gas exchange systems require several to tens of minutes to acclimatize a leaf to the leaf chamber, limiting the number of samples to be examined^41^. To overcome this limitation, we recently created a new closed gas exchange system (MIC-100; Masa International Corporation, Kyoto, Japan), which takes 15–20 s per measurement, ~ 90% less than conventional open gas exchange systems. We hypothesize that with the new measurement system, tracing photosynthetic dynamics of multiple rice accessions across their growing season will tell us which photosynthetic dynamics can maximize productivity and which developmental stage should be targeted in breeding for photosynthesis.

In previous studies, our research group determined that the *indica* cultivar Takanari, which has one of the highest grain yields among Japanese rice cultivars, accumulated more biomass than Nipponbare and Koshihikari, standard *japonica* cultivars^42,43^. Since then, Takanari has been widely used to analyse the physiological and molecular mechanisms of biomass accumulation^44–50^ and their effects on grain yield^51–54^. Although the higher biomass accumulation in Takanari is characterized by a higher net assimilation rate around the full heading stage, which could be partly explained by the higher leaf photosynthetic capacity, only rough analysis of gas exchange during growth has been conducted^43^. Here, we aimed at collecting the data on temporal changes in canopy photosynthesis of Koshihikari and Takanari over the entire growing season by using the MIC-100 to analyse its association with crop growth rate (CGR) and total biomass accumulation. We assumed that photosynthesis in the uppermost fully expanded leaf is representative of canopy photosynthesis, since it has the highest photosynthetic capacity and receives the strongest radiation in the canopy^43,46,55^. We also observed ontogenic changes of chlorophyll content (SPAD value) and single leaf area (single LA). To analyse the phenotypic variation caused by introgressions between the cultivars, we used reciprocal sets of chromosome segment substitution lines (reciprocal CSSLs) derived from a Koshihikari/Takanari cross^52,56^. Each CSSL carries a single genomic segment from the donor cultivar (either Koshihikari or Takanari) in the genetic background of the other cultivar, and the full set of substituted segments covers the entire genome^52,57^. The variation in flowering date is much smaller in CSSLs than in other populations such as recombinant inbred lines, which is advantageous in examining whether changes in photosynthesis affect biomass accumulation. From this study, we propose that maintaining a high rate of photosynthesis after heading, rather than having a high maximum photosynthetic rate, can increase total biomass accumulation.

## Results

### Ontogenic changes in photosynthesis and biomass accumulation

**Figure 1:**
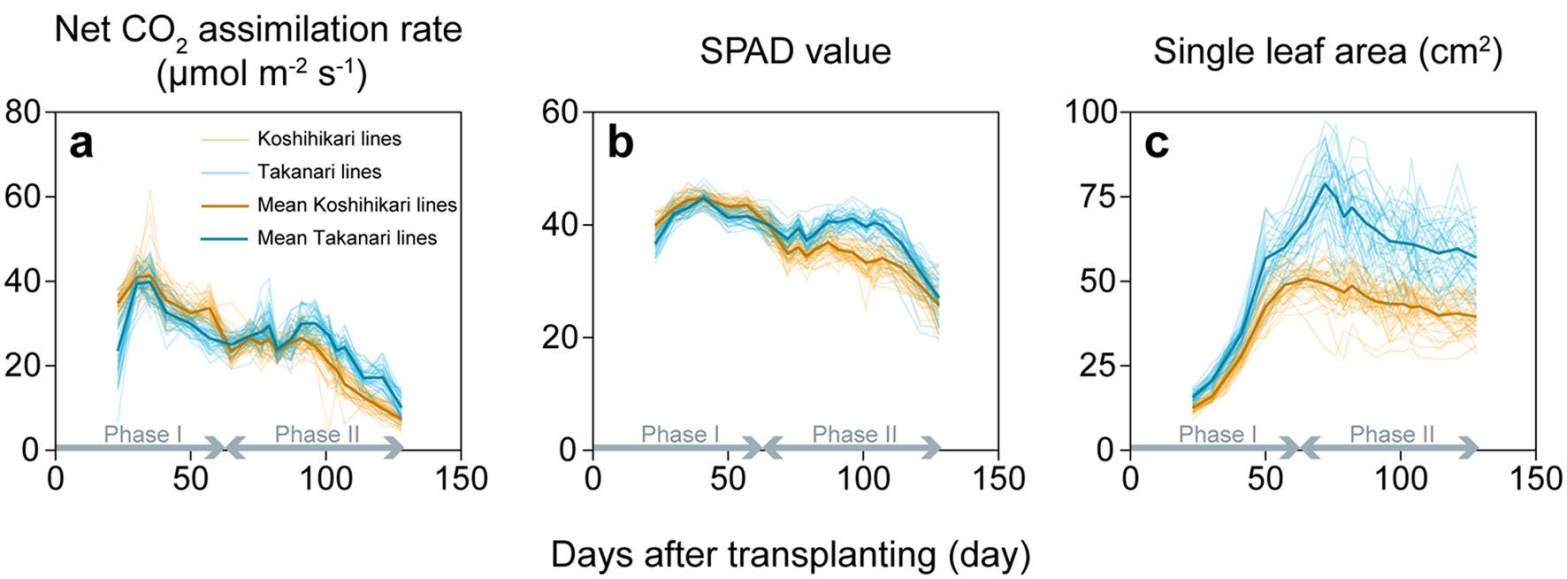
Dynamics of (**a**) net CO_2_ assimilation rate, (**b**) SPAD value and (**c**) single leaf area across the entire growing season. Koshihikari lines, Koshihikari-background CSSLs and Koshihikari; Takanari lines, Takanari-background CSSLs and Takanari (*n* = 3). Mean phenotypic values are also shown. Phase I, transplanting to first biomass sampling (71 days after transplanting, DAT); Phase II, first biomass sampling to second biomass sampling (129 DAT).

We divided the growth period into Phase I—from transplanting to the first biomass sampling (at heading)—and Phase II—from the first sampling to the second sampling (at harvest) (Fig. 1). (See days to heading [DTH] data of all rice lines in Supplementary Dataset S1.) As a general trend, *A* reached the maximum at around 30–35 days after transplanting (DAT) and then gradually decreased over time (Fig. 1a). During Phase I, *A* values of Takanari-background CSSLs and Takanari (Takanari lines) were lower by 10% in average than those of Koshihikari-background CSSLs and Koshihikari (Koshihikari lines). During Phase II, *A* values of Takanari lines remained higher than those of Koshihikari lines (Fig. 1a). SPAD values showed a similar trend (Fig. 1b). Single LA gradually increased with crop growth and reached a maximum at around 65 DAT in Koshihikari lines and 72 DAT in Takanari lines (Fig. 1c). Single LA of Takanari lines was larger than that of Koshihikari lines during Phase I, and larger still during Phase II (Fig. 1c).

**Figure 2:**
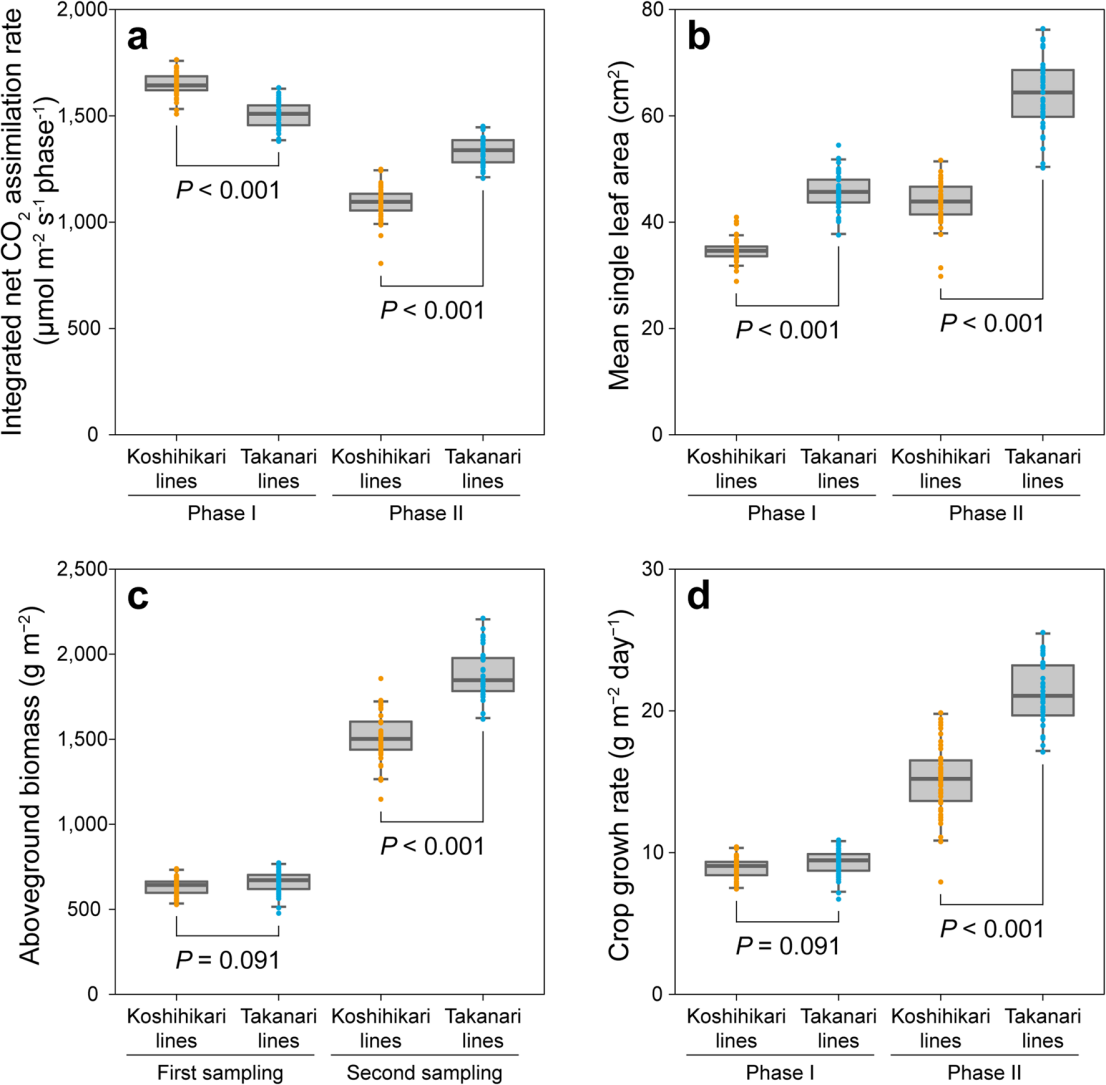
Comparisons of phenotypes between Koshihikari lines and Takanari lines. (**a**) Integrated net CO_2_ assimilation rate, (**b**) mean single leaf area, (**c**) dry weight of aboveground biomass, (**d**) crop growth rate. Abbreviations as in Fig. 1. Boxplots: central line, median; boxes, interquartile range (IQR); whiskers, 1.5 × IQR; points, actual data.

Integrated *A* (*A*_int_), the apparent total CO_2_ uptake calculated by sum of trapezoidal area under each pair of adjacent measurements, was 10% lower in Takanari lines than in Koshihikari lines during Phase I, but was 23% higher during Phase II (*P* < 0.001; Fig. 2a). Mean single LA was significantly higher in Takanari lines than in Koshihikari lines during Phase I, and even higher during Phase II (*P* < 0.001; Fig. 2b). There was no significant difference in aboveground biomass at the first sampling (AGB_ I_) or in CGR during Phase I (CGR _Phase I_) between Koshihikari lines and Takanari lines, while AGB at the second sampling (AGB _II_) and CGR during Phase II (CGR _Phase II_) were significantly higher in Takanari lines than in Koshihikari lines, by 25% and 40%, respectively (*P* < 0.001; Fig. 2c,d). The standard deviation (SD) in each background was larger during Phase II than during Phase I (for instance, for CGR in Koshihikari lines: 0.72 during Phase I but 2.51 during Phase II; Supplementary Table S1). These results indicate that the genetic differences between Koshihikari and Takanari and between lines of each genetic background were more notable during Phase II than during Phase I. The AGB _II_ was closely correlated with CGR _Phase II_ (*r* = 0.97), not with CGR _Phase I_ (*r* = 0.34; Supplementary Fig. S4). CGR _Phase I_ was not correlated with *A*_int_ during Phase I (*A*_int Phase I_) (*r* = − 0.10) and was only slightly correlated with mean single LA during Phase I (LA_mean Phase I_) (*r* = 0.28), while CGR _Phase II_ was strongly correlated with these values (*r* = 0.75 for *A*_int_ during Phase II [*A*_int Phase II_], *r* = 0.82 for mean single LA during Phase II [LA_mean Phase II_]; Supplementary Fig. S4). These results indicate that AGB _II_ depends largely on CGR _Phase II_, which in turn is correlated closely with photosynthesis and single LA during Phase II.

### Curve-smoothing analysis during Phase II and associations of parameters with crop growth rate

**Figure 3:**
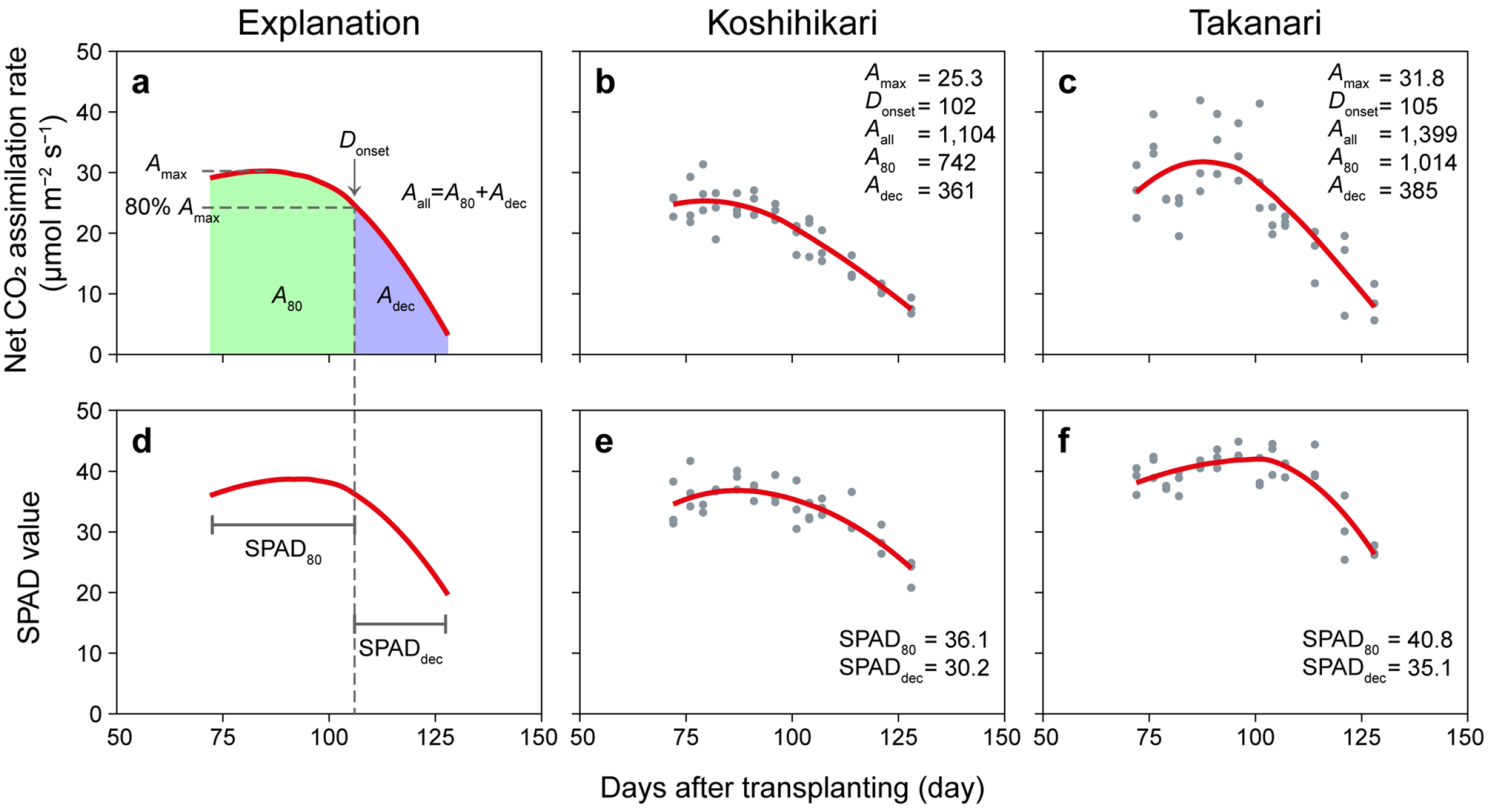
Curve-smoothing analysis for net CO_2_ assimilation rates (*A*) and SPAD values during Phase II. (**a**) *A* curve. *A*_max_, maximum fitted value of *A*; *D*_onset_, 1 day before *A* declined below 80% of *A*_max_; *A*_all_, accumulated *A* during Phase II; *A*_80_, accumulated *A* from 72 DAT to *D*_onset_; *A*_dec_, accumulated *A* from *D*_onset_ to 128 DAT. (**b**,**c**) Curve-smoothing analysis of *A* for (**b**) Koshihikari and (**c**) Takanari. Grey points, actual data; red lines, smoothed curves. (**d**) SPAD curve. SPAD_80_, mean SPAD value before *D*_onset_; SPAD_dec_, mean SPAD value after *D*_onset_. (**e**,**f**) Curve-smoothing analysis of SPAD for (**e**) Koshihikari and (**f**) Takanari.

For detailed analysis of photosynthetic dynamics during Phase II, we applied curve-smoothing analysis to the *A* and SPAD values (Fig. 3a,d). Both curves were upward-convex, peaking several days after beginning of Phase II, and decreased over time. The total area under the curve (*A*_all_) and the maximum *A* (*A*_max_) were higher in Takanari than in Koshihikari, by around 26% each (Fig. 3b,c). When *A*_all_ was divided into accumulated *A* at > 80% of *A*_max_ (*A*_80_) and accumulated *A* at ≤ 80% of *A*_max_ (*A*_dec_) at *D*_onset_ (1 day before *A* declined below 80% of *A*_max_), Takanari had a higher *A*_80_ than Koshihikari but a similar *A*_dec_ (Fig. 3b,c). Takanari also had higher values of SPAD_80_ and SPAD_dec_ (the mean SPAD values of the two phases divided at *D*_onset_) than Koshihikari (Fig. 3e,f). The values of all CSSLs are shown in the Supplementary Dataset S1.

**Figure 4:**
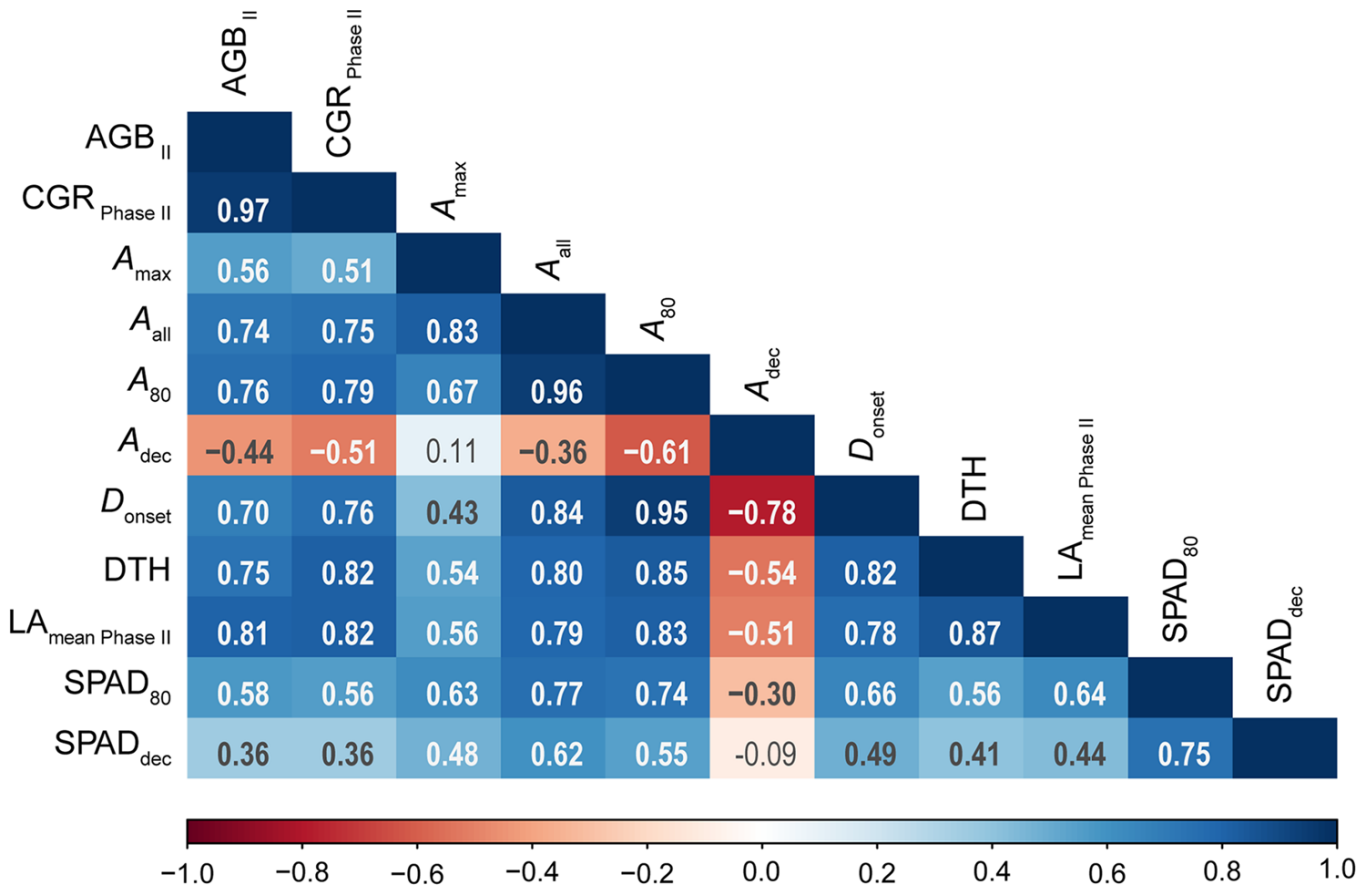
Pearson’s correlation coefficients of pairs of traits (biomass accumulation, CO_2_ assimilation and other agronomic traits) during Phase II among all lines examined. Values in bold type are significant (*P* < 0.05, two-sided *t*-test). Blue, positive correlation; red, negative correlation. AGB _II_, dry weight of aboveground biomass harvested at 128 DAT; CGR _Phase II_, crop growth rate during Phase II; DTH, days to heading; LA_mean Phase II_, mean value of single leaf area during Phase II. Other abbreviations as in Fig. 3.

The correlations between biomass accumulation and photosynthetic parameters after heading showed that CGR _Phase II_ was closely correlated with *A*_all_ (Fig. 4). In turn, *A*_all_ was closely correlated with *A*_max_, *A*_80_ and *D*_onset_, and was moderately negatively correlated with *A*_dec_. These results suggest that *A*_all_ is determined mainly by *A*_80_, the magnitude of which can be explained by both *A*_max_ and *D*_onset_. CGR _Phase II_ was positively correlated with DTH and LA_mean Phase II_, indicating that a later heading date and a larger single LA could enhance biomass accumulation. SPAD_80_ was positively correlated with *A*_80_, but SPAD_dec_ was not correlated with *A*_dec_.

### Analysis by genetic background

**Figure 5:**
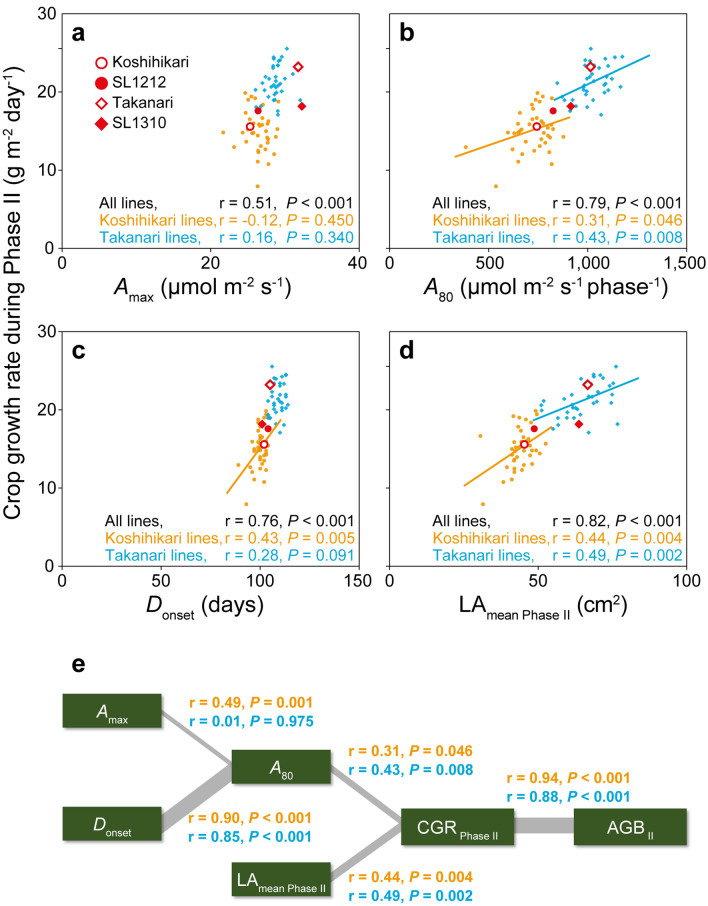
Relationships between CGR _Phase II_ and (**a**) *A*_max_, (**b**) *A*_80_, (**c**) *D*_onset_ and (**d**) LA_mean Phase II_. Orange, Koshihikari lines; blue, Takanari lines. (**e**) Schematic model showing the factors affecting biomass accumulation. Abbreviations as in Figs. 3 By multiple linear regression analysis, the combined contribution of *A*_80_ and LA_mean Phase II_ to CGR _Phase II_ variation and 4.

The results of the above analyses should be affected considerably by the genetic background, because the Takanari lines had consistently higher values of most parameters after heading. So we conducted separate analyses by genetic background. AGB _II_ was closely correlated with CGR _Phase II_ in each background (*r* = 0.94 for Koshihikari lines, *r* = 0.88 for Takanari lines; Supplementary Fig. S5). CGR _Phase II_ was not correlated with *A*_max_ in either background (*r* = − 0.12 for Koshihikari lines, *r* = 0.16 for Takanari lines), but it was significantly correlated with *A*_80_ (*r* = 0.31 for Koshihikari lines; *r* = 0.43 for Takanari lines) and with *D*_onset_ (*r* = 0.43 for Koshihikari lines; Supplementary Fig. S5, Fig. 5a–c). The association between CGR _Phase II_ and *D*_onset_ in Takanari lines was close to significant (*r* = 0.28, *P* = 0.091; Supplementary Fig. S5b, Fig. 5c). These results indicate that maintaining a high rate of photosynthesis for longer, rather than having a higher *A*_max_, was related to higher biomass accumulation during Phase II in each background. We also found a significant relationship between CGR _Phase II_ and LA_mean Phase II_ in each background (*r* = 0.44 for Koshihikari lines, *r* = 0.49 for Takanari lines; Supplementary Fig. S5, Fig. 5d). The factors affecting total biomass accumulation are presented in Fig. 5e. was 25% for Koshihikari lines and 31% for Takanari lines.

Among the CSSLs, *A*_80_ values of SL1212 and SL1310, with a single genomic segment on chromosome 3 from the introgression parent, were, respectively, 11% higher than that of Koshihikari and 10% lower than that of Takanari (Supplementary Fig. S6b). SPAD_80_ was similar between each pair of the parent and the CSSL, suggesting less difference in biochemical properties (Supplementary Fig. S6c). AGB _II_ and CGR _Phase II_ of SL1212 were 13% and 13%, respectively, higher than those of Koshihikari, and those of SL1310 were 14% and 28% lower than those of Takanari (Supplementary Fig. S6d,e). These results suggest that genes on the genomic region of chromosome 3 (17.0–29.2 Mb) regulate both photosynthesis and total biomass accumulation. We also found some yield-related genes included in this genomic region (Supplementary Table S2; Supplementary Dataset S2)^58^.

## Discussion

Improving leaf photosynthetic capacity has long been considered a promising target to increase biomass production and yield in crop species^6,9,10^. However, poor correlations between leaf photosynthetic rate and biomass accumulation or yield have been reported, perhaps in part owing to limited datasets^18,21,31,32,59^. To understand the association of photosynthetic rate and biomass accumulation across the entire growing season, we obtained high-frequency data of *A*, SPAD and single LA of the canopy leaf and tested correlations with CGR using reciprocal CSSLs and their parental cultivars.

During Phase I (transplanting to heading), differences in leaf photosynthesis had little effect on biomass production. Takanari lines had lower *A* and SPAD values and larger single LA than Koshihikari lines (Figs. 1, 2). AGB_ I_ and CGR _Phase I_ did not differ between Koshihikari and Takanari lines (Fig. 2), which can be explained by the offset of the lower *A* by the larger single LA in Takanari lines. Taylaran et al. likewise showed that Takanari had a similar plant growth rate to Koshihikari during the vegetative stage owing to its lower net assimilation rate but the higher mean leaf area per plant^43^. We also found a smaller variation in these traits among lines of each background (41 Koshihikari lines, 37 Takanari lines) during Phase I than during Phase II (Fig. 2; Supplementary Table S1), which suggests that the genomic introgressions between the cultivars have little effect on phenotypic expression before heading. In contrast, a wide genetic variation in biomass accumulation (227%) among 204 global mini-core accessions and 11 elite Chinese rice cultivars at the mid-vegetative stage (60 days after emergence) was reported^19^. The authors also showed that *A* under low light was highly related to biomass accumulation, suggesting that simultaneous improvements of photosynthetic rate and biomass accumulation during early growth can be achieved by using a diverse set of germplasms^19^.

During Phase II (heading to harvest), photosynthetic parameters were closely associated with biomass accumulation. The *A* value of Takanari lines increased and remained higher than that of Koshihikari lines until the final examination (Fig. 1a). The positive correlations between photosynthetic parameters (*A*_max_, *A*_all_, *A*_80_) and biomass accumulation parameters (CGR _Phase II_ and AGB _II_) in the analysis of all datasets combined indicate that the consistently higher photosynthetic capacity in Takanari lines contributes to the enhanced biomass accumulation compared to Koshihikari lines (Fig. 4). The large difference in *A* between Koshihikari and Takanari after heading stage has been repeatedly reported^43–45,48^. This is explained in part by the enhanced root system development in Takanari, increasing water and nitrogen uptake^43^. Additionally, the steeper nitrogen distribution to the upper canopy leaf in Takanari can lead to the effective use of sunlight^6^. In the separate analysis of each background, CGR _Phase II_ and *A*_80_ were significantly correlated in both Koshihikari and Takanari lines (Supplementary Fig. S5). Interestingly, in this analysis, *A*_max_ was not correlated with CGR _Phase II_ in either Koshihikari or Takanari lines (Fig. 5a, Supplementary Fig. S5). In addition, *A*_max_ was not correlated with *A*_80_ in Takanari lines, although it was significantly correlated in Koshihikari lines (Supplementary Fig. S5), suggesting that increasing *A*_max_ is not always an efficient strategy for enhancing biomass accumulation. Many physiological and molecular analyses have focused on the maximum photosynthetic rate of the flag leaf on the assumption that it has the highest photosynthetic activity in the crop canopy after heading, which would be closely correlated with biomass accumulation and yield^26,44,56,60,61^. However, our results show that maintaining a high rate of photosynthesis after heading, rather than having a high *A*_max_, is more closely associated with biomass accumulation. We identified a genomic region that may simultaneously increase (or decrease) *A*_80_ and CGR _Phase II_ without increasing *A*_max_ (Fig. 5, Supplementary Fig. S6). We propose that as limited evaluation of photosynthesis could select lines with poor biomass accumulation, ontogenic changes in photosynthesis after heading should be examined for simultaneously enhancing photosynthetic performance and biomass accumulation.

The variation in LA_mean Phase II_ was also associated with the variation in CGR _Phase II_ in both the combined analysis of all lines and the separate analyses of Koshihikari and Takanari lines (Figs. 4, 5d, Supplementary Fig. S5). This suggests that breeding for the selection of plants with larger flag leaves may enhance the total biomass accumulation. However, very large leaves and too many tillers could reduce sunlight penetration into the canopy, thereby increasing canopy respiration rate and decreasing the total biomass accumulation^62^. This issue may not be obvious in our results, but it should be considered in different growth environments.

Although we found significant correlations of *A*_80_ and LA_mean Phase II_ with CGR _Phase II_, the combined contribution of *A*_80_ and LA_mean Phase II_ to CGR _Phase II_ variation was only 25–31%. This indicates the presence of other major determinants behind the variation in CGR. The first possibility is tiller growth: active tillering increases the total leaf area of a plant, greatly contributing to total biomass accumulation and panicle number^63^. Although we did not examine tiller numbers here, panicle number varies widely among these CSSLs^52^. The second possibility is the photosynthetic capacity and single LA of leaves at lower positions. A recent study showed that the balance of photosynthetic capacity between the flag leaf and the leaf immediately below it has significant effects on canopy photosynthesis in wheat^64^. The third possibility is the degree of light penetration to the bottom of the canopy. Better light penetration, which is achieved by large leaf inclination angles and decreased chlorophyll content of the canopy leaves, can maximize canopy photosynthesis^6,65^. Takanari has one of the highest leaf inclination angles among rice cultivars, which is considered an important determinant of its higher biomass accumulation^66^. The fourth possibility is adaptation to the environment, especially light and vapour pressure deficit. Sunlight reaching the leaf surface fluctuates on the order of minutes to seconds owing to cloud, wind and self-shading^67^. The time-lag inherent in reaching a new steady-state rate of photosynthesis after a fluctuation would diminish the total carbon gain^68,69^. The photosynthetic rate can decrease in the afternoon on sunny days with high vapour pressure deficit, so-called “midday depression”, largely because of closed stomata and photoinhibition^3,70^. So we need a comprehensive simulation model using these complex physiological factors and the association of the underlying genomic regions to explain the difference in biomass accumulation among lines. Our high-frequency datasets of photosynthesis may contribute to the development of such a model.

In conclusion, maintaining a higher photosynthetic rate, rather than achieving the maximum rate, after heading was closely associated with biomass accumulation. We identified a genomic region likely to simultaneously increase *A*_80_ and biomass accumulation, although further investigation is necessary. We propose that examination of the dynamics of photosynthesis throughout the entire growing period is important to the use of natural genetic resources for breeding selection. In contrast, the limited contribution of *A*_80_ to biomass production suggests essential roles of other physiological factors in biomass variation. A comprehensive model explaining the role of genetic variation in biomass accumulation by multiple physiological properties and the roles of key genes is required.

## Methods

### Plant cultivation

Collection of plant material, must comply with relevant institutional, national, and international guidelines and legislation. The rice seeds of Koshihikari and Takanari and reciprocal sets of CSSLs (41 lines in Koshihikari background, 39 lines in Takanari background)^52^ were obtained from Institute of Crop Science, National Agriculture and Food Research Organization, Tsukuba, Japan, with a material transfer agreement. Lines SL1208, SL1335 and SL1336 had a dwarf plant structure, probably due to hybrid breakdown associated with the interaction of *hbd2* and *hbd3*, and SL1320 did not produce panicles during the experiment, probably owing to the inserted *Hd1* gene^52^. We excluded these four lines from our analyses. Seeds were sown in plastic cups filled with artificial soil on 7 May 2019, and the seedlings were grown until the fourth to fifth leaf stage in the greenhouse. They were transplanted into a paddy field (an alluvial clay loam) of Tokyo University of Agriculture and Technology (35° 39′ N, 139° 28′ E) on 22 May with a basal dressing of inorganic fertilizer supplying 30 kg N, 60 kg P, and 60 kg K ha^−1^. One-third of the total N was applied as ammonium sulphate, and the other two-thirds as slow-release urea (LP-50 & LPS-100; JCAM Agri Co., Ltd, Tokyo, Japan). No topdressing was applied. The plant density was 22.2 m^−2^ (at a spacing of 30 cm × 15 cm) with one plant per hill, and plants were grown under submerged conditions. Each line was grown in three replicate plots in 2 rows of 20 plants (60 cm × 300 cm). Plots were randomized, but lines of each background group were planted adjacent (Supplementary Fig. S1).

### Phenotypic analysis

The uppermost newly expanded leaf on the main tiller of one plant per plot was used for phenotypic analyses. The net CO_2_ assimilation rate was measured with a closed-type portable photosynthesis system (MIC-100; Masa International Corporation, Kyoto, Japan; https://www.weather.co.jp/catalog_html/MIC-100.html), which consists of a console and a chamber head with an aperture area of 2 cm × 3 cm (Supplementary Fig. S2a). A non-dispersive infrared sensor at the bottom of the chamber measures CO_2_ concentration every 0.1 s. To prevent rapid inactivation of the leaf’s photosynthetic activity, a light-emitting diode lamp at the chamber top supplies a photosynthetic photon flux density of 1200 μmol photons m^−2^ s^−1^. After an intact leaf is enclosed in the chamber clip, air flow from the atmosphere is blocked off, and the rate of decrease of CO_2_ concentration from 400 to 390 ppm is monitored to calculate net CO_2_ assimilation rate. Each measurement was completed within 20 s. Measurements were taken in sunlight between 08:00 and 13:00 h on dry days (the solar radiation during measurements was 500–1300 μmol photons m^−2^ s^−1^). The SPAD value as a proxy for leaf chlorophyll content was measured with a chlorophyll meter (SPAD-502; Konica Minolta, Osaka, Japan; Supplementary Fig. S2b). Leaves were sampled and transported to the laboratory without dehydration. The leaves put in a transparent folder were passed through a commercial document scanner (ScanSnap iX1500; Fujitsu, Kanagawa, Japan; Supplementary Fig. S2c). The single LA and the partial LA in the MIC-100 chamber were measured in ImageJ software (National Institutes of Health, Bethesda, MD, USA). The partial LA was used for the calculation of net CO_2_ assimilation rate per leaf area (*A*, μmol m^−2^ s^−1^). Measurements were conducted once a week from 3 weeks after transplanting (14 June, 23 DAT) to harvest (27 September, 128 DAT), and additional measurements were also conducted around heading and mid-ripening stage (20 days in total). In total, 246 leaves were measured per day, 4632 leaves during the experiment. *A*_int_ was calculated by summing the trapezoidal area under each pair of adjacent measurement cycles. Mean single LA was calculated as the average of linear interpolated values.

### Sampling of aboveground biomass

The aboveground biomass was examined at heading (1 August, 71 DAT; AGB_ I_) and harvest (28 September, 129 DAT; AGB _II_). Eight plants in each plot were sampled and air-dried in a greenhouse until weighing. Air-dried samples of parental plants were dried in a ventilated oven to calculate the water content ratio of the air-dried samples. The biomass accumulation was expressed as dry weight (g) per m^2^. CGR from transplanting (considered as 0) to heading (Phase I) and from heading to harvest (Phase II) were calculated.

### Curve smoothing and statistical analysis

All statistical analyses were performed in R v. 4.0.2 software^71^. The changes in *A* and SPAD values during Phase II in each rice line were smoothed by the Locally Weighted Scatterplot Smoother (LOESS) algorithm with the smoothing parameter fixed at 1.0^72^. We defined *A*_max_ (μmol m^−2^ s^−1^) as the maximum fitted value of *A*, and *D*_onset_ (day) as 1 day before *A* declined below 80% of *A*_max_ (Fig. 3a). We also defined *A*_all_ (μmol m^−2^ s^−1^ phase^−1^) as accumulated *A* by curve-smoothing during Phase II (from 72 to 128 DAT), *A*_80_ (μmol m^−2^ s^−1^ phase^−1^) as accumulated *A* from 72 DAT to *D*_onset_, and *A*_dec_ (μmol m^−2^ s^−1^ phase^−1^) as accumulated *A* from *D*_onset_ to 128 DAT (Fig. 3a). We show the changes in correlation coefficients between CGR _Phase II_ and *A*_10_ to *A*_90_ (accumulated *A* at > 10% to > 90% of *A*_max_, Supplementary Fig. S7). SPAD_80_ is the mean SPAD value from 72 DAT to *D*_onset_, and SPAD_dec_ is mean SPAD value from *D*_onset_ to 128 DAT. Statistical differences were tested by Welch’s two-sided *t*-test. Pearson’s correlation coefficient was calculated, and the significance of relationships was tested by two-sided *t*-tests.

## Supplementary Information

Supplementary Dataset S1.

Supplementary Dataset S2.

Supplementary Information.

## Data Availability

All data and plant materials are available from the corresponding author on reasonable request.

## Abbreviations

*A*: Net CO_2_ assimilation rate per leaf area
*A*_80_: Accumulated *A* at > 80% of *A*_max_
*A*_all_: Accumulated *A* during Phase II
*A*_dec_: Accumulated *A* at ≤ 80% of *A*_max_
AGB_ I_: Aboveground biomass at first sampling
AGB_II_: Aboveground biomass at second sampling
*A*_int_: Integrated *A*
*A*_int Phase I_: Integrated *A* during Phase I
*A*_int Phase II_: Integrated *A* during Phase II
*A*_max_: Maximum fitted value of *A* during Phase II
CGR: Crop growth rate
CGR_Phase__I_: CGR during Phase I
CGR_Phase__II_: CGR during Phase II
CSSL: Chromosome segment substitution line
DAT: Days after transplanting
*D*_onset_: 1 Day before *A* declined below 80% of *A*_max_
DTH: Days to heading
Koshihikari lines: Koshihikari-background CSSLs and Koshihikari
LA: Leaf area
LA_mean Phase I_: Mean single LA during Phase I
LA_mean Phase II_: Mean single LA during Phase II
SPAD_80_: Mean SPAD value before *D*_onset_
SPAD_dec_: Mean SPAD value after *D*_onset_
Takanari lines: Takanari-background CSSLs and Takanari
