# Supplementary material for: Maintaining higher leaf photosynthesis after heading stage could promote biomass accumulation in rice
Source: Sci Rep. 2021 Apr 7;11:7579. doi: 10.1038/s41598-021-86983-9 (PMC8027620; doi:10.1038/s41598-021-86983-9)
Supplement: Supplementary file 3 — Supplementary Information. [file 41598_2021_86983_MOESM3_ESM.pdf]

Supplementary Material for

Maintaining higher leaf photosynthesis after heading stage could promote biomass accumulation in rice

Sotaro Honda, Satoshi Ohkubo, Nan Su San, Anothai Nakkasame, Kazuki Tomisawa, Keisuke Katsura, Taiichiro Ookawa, Atsushi J. Nagano, Shunsuke Adachi*

*Corresponding author: S. Adachi, shunsuke.adachi.0210@vc.ibaraki.ac.jp

The PDF includes:

Supplementary Figures S1 to S7

Supplementary Tables S1 and S2

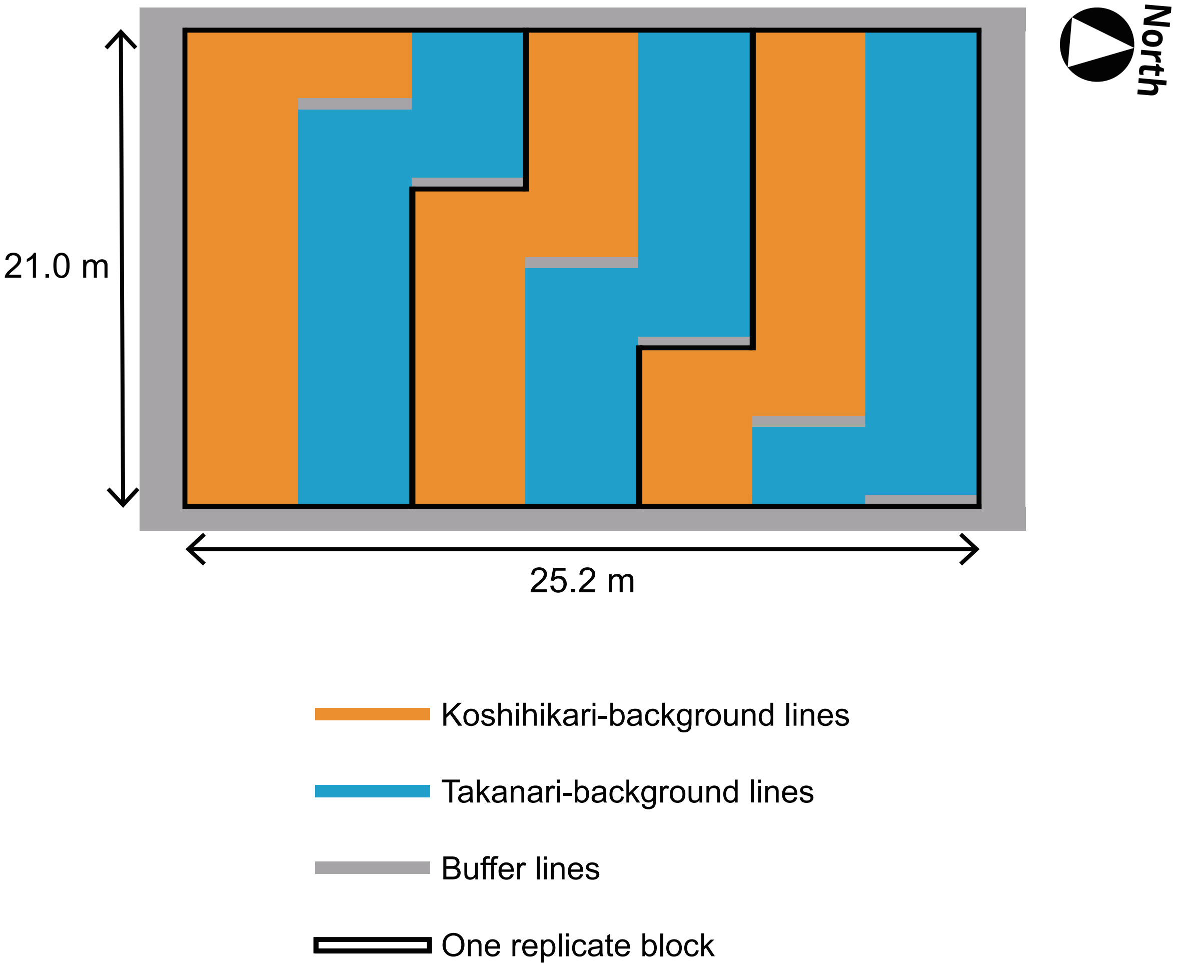

Supplementary Figure S1. Layout of the experimental plots in the paddy field.

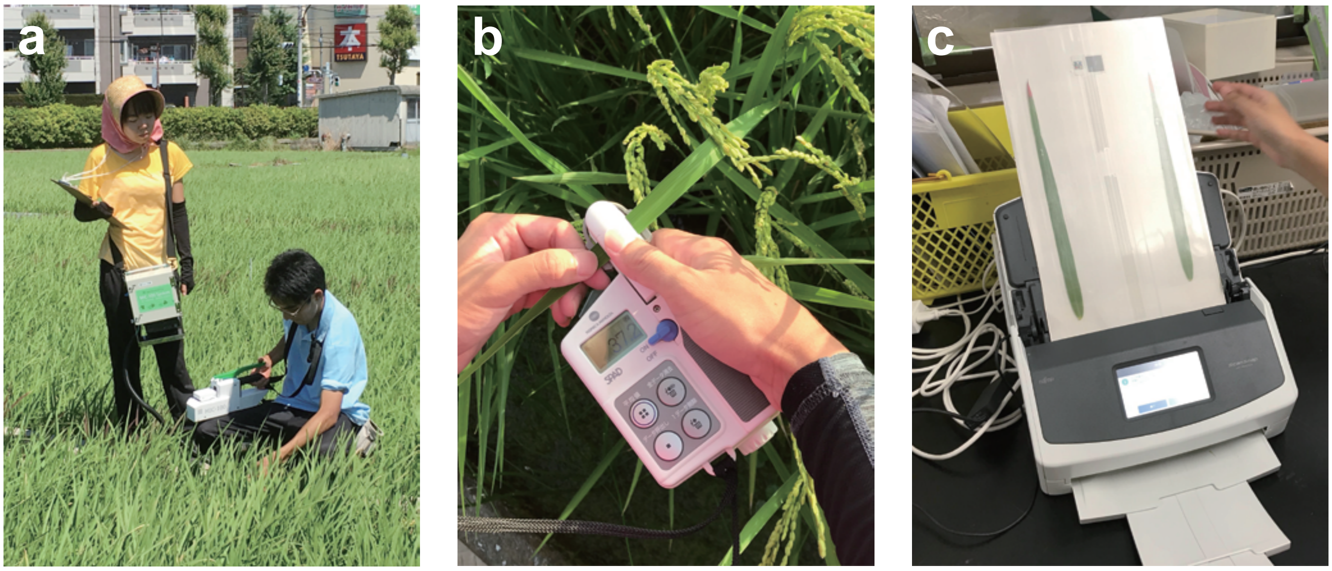

Supplementary Figure S2. Phenotyping procedures.
(**a**) Gas exchange measurement with a MIC-100 closed-type portable photosynthesis system.
(**b**) SPAD measurement with a SPAD-502 chlorophyll meter.
(**c**) Scanning of leaves with a ScanSnap iX1500 document scanner.

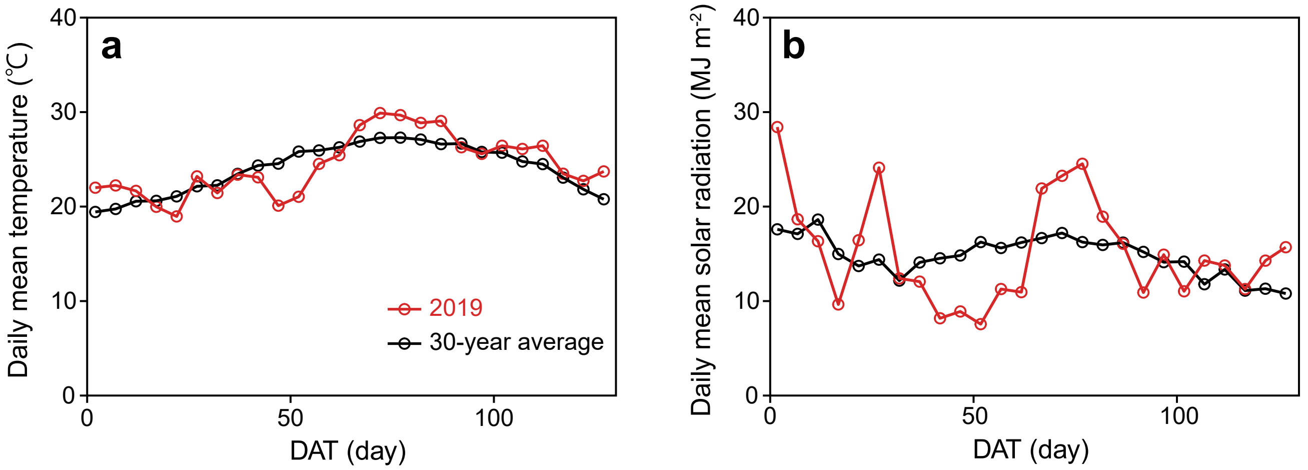

Supplementary Figure S3. Dynamics of (**a**) daily mean temperature and (**b**) daily mean solar radiation during the growing season. Daily mean temperature was obtained from the Automated Meteorological Data Acquisition Systems (AMeDAS) Fuchu site (35°41′N, 139°29′E) and daily mean global solar radiation was obtained from the AMeDAS Tokyo site (35°42′N, 139°45′E). The points are 5-day averages. DAT, days after transplanting.

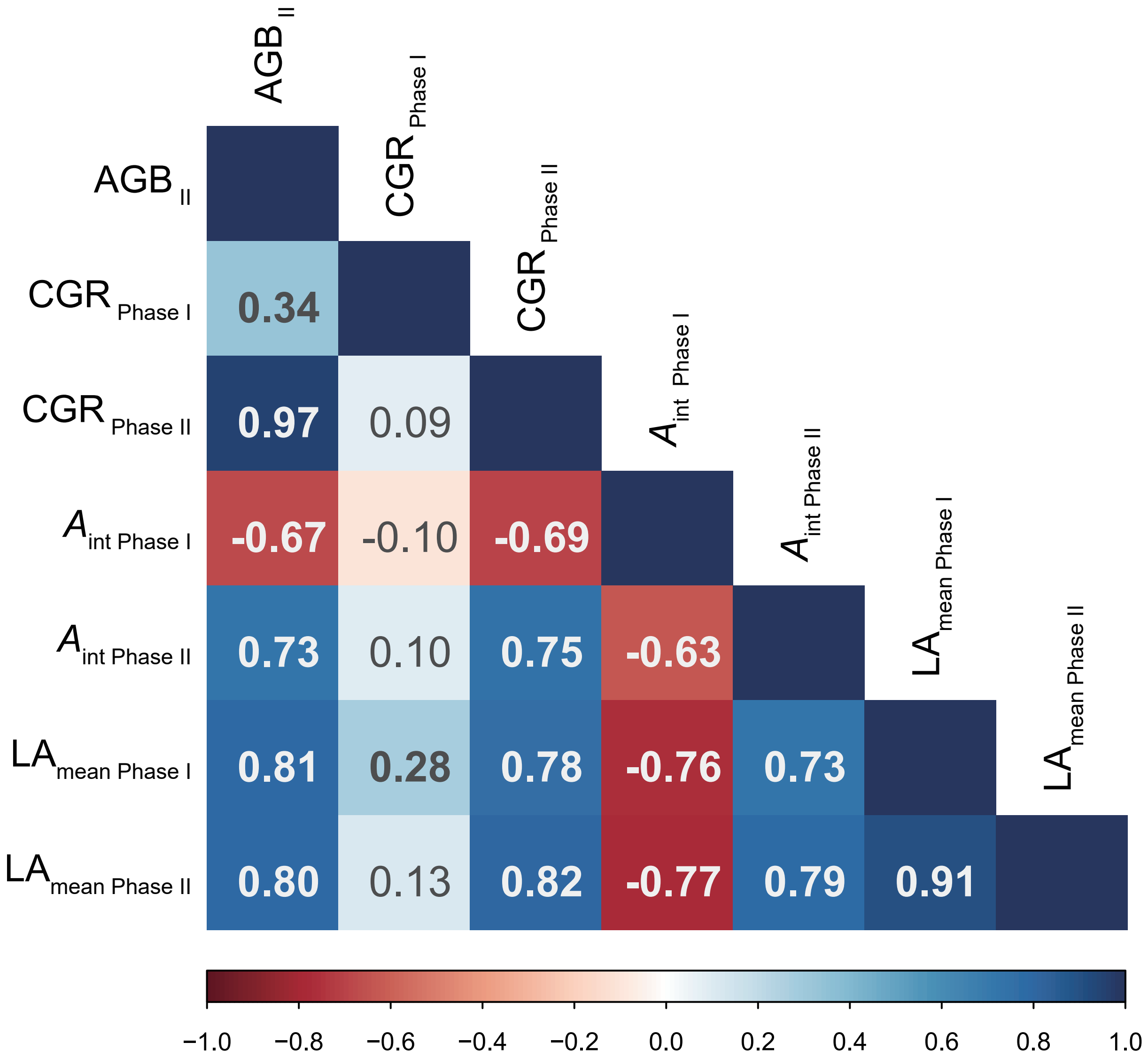

Supplementary Figure S4. Pearson's correlation coefficients of pairs of traits (biomass, crop growth rate, integrated CO_2 assimilation rate and mean single leaf area) among all lines examined. Values in bold type are significant ($P < 0.05$, two-sided *t*-test). Blue, positive correlation; red, negative correlation. AGB_{II}, dry weight of aboveground biomass at the second sampling; $CGR_{Phase\ I}$ and $CGR_{Phase\ II}$, crop growth rate during Phases I and II; $A_{int\ Phase\ I}$ and $A_{int\ Phase\ II}$, integrated *A* during Phases I and II; $LA_{mean\ Phase\ I}$ and $LA_{mean\ Phase\ II}$, mean single leaf area during Phases I and II.

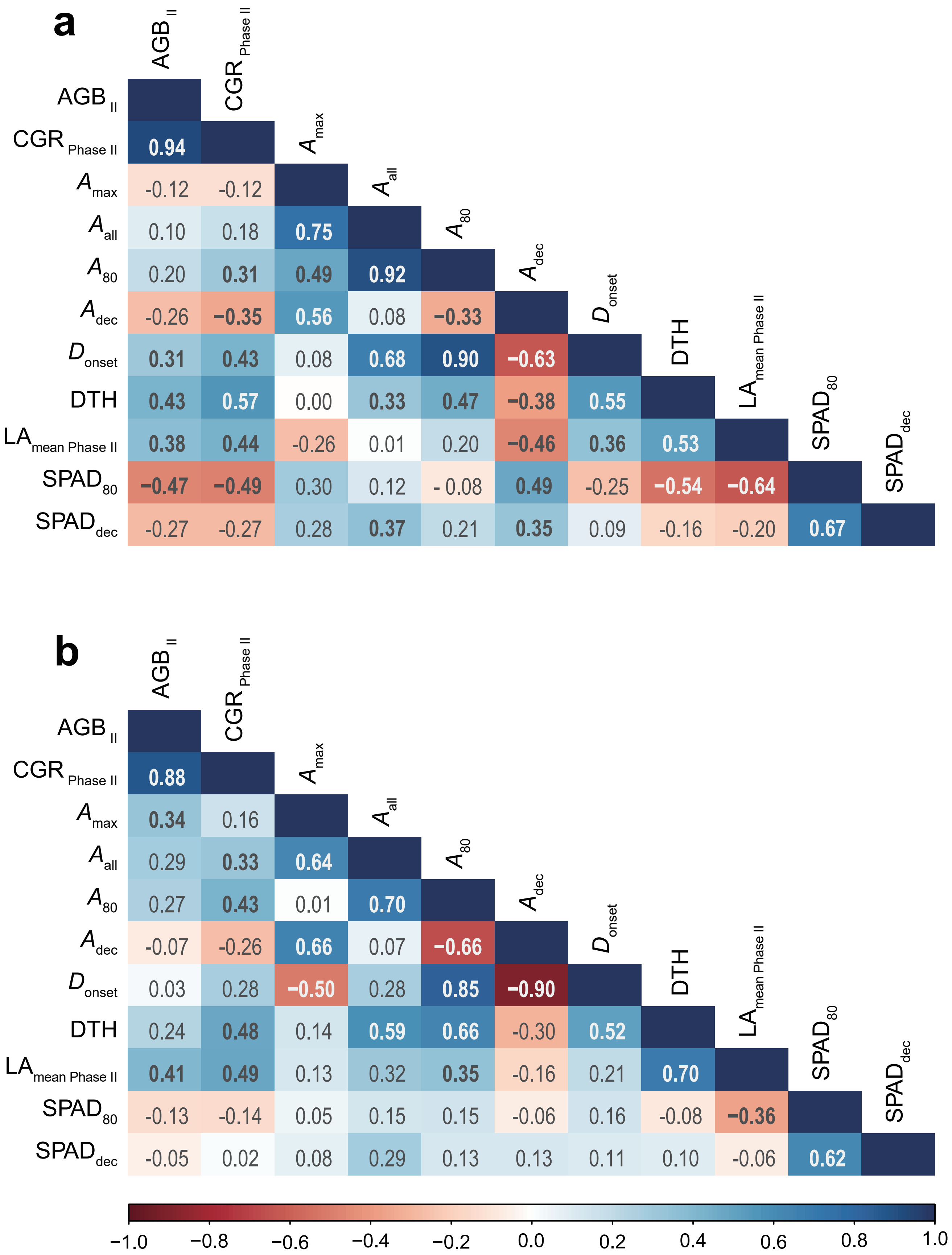

Supplementary Figure S5. Pearson's correlation coefficients of pairs of traits (biomass accumulation, CO_2 assimilation rate and other agronomic traits) in (**a**) Koshihikari lines and (**b**) Takanari lines during Phase II. Values in bold type are significant (P < 0.05, two-sided t-test). Blue, positive correlation; red, negative correlation. AGB_{II}, dry weight of aboveground biomass at the second sampling; $CGR_{Phase\ II}$, crop growth rate during Phase II; A_{max}, maximum fitted value of A; A_{all}, accumulated A during Phase II; A_{80}, accumulated A from 72 days after transplanting (DAT) to D_{onset}; A_{dec}, accumulated A from D_{onset} to 128 DAT; D_{onset}, 1 day before A value declined below 80% of A_{max}; DTH, days to heading; $LA_{mean\ Phase\ II}$, mean value of single leaf area during Phase II; $SPAD_{80}$, mean SPAD value before D_{onset}; $SPAD_{dec}$, mean SPAD value after D_{onset}.

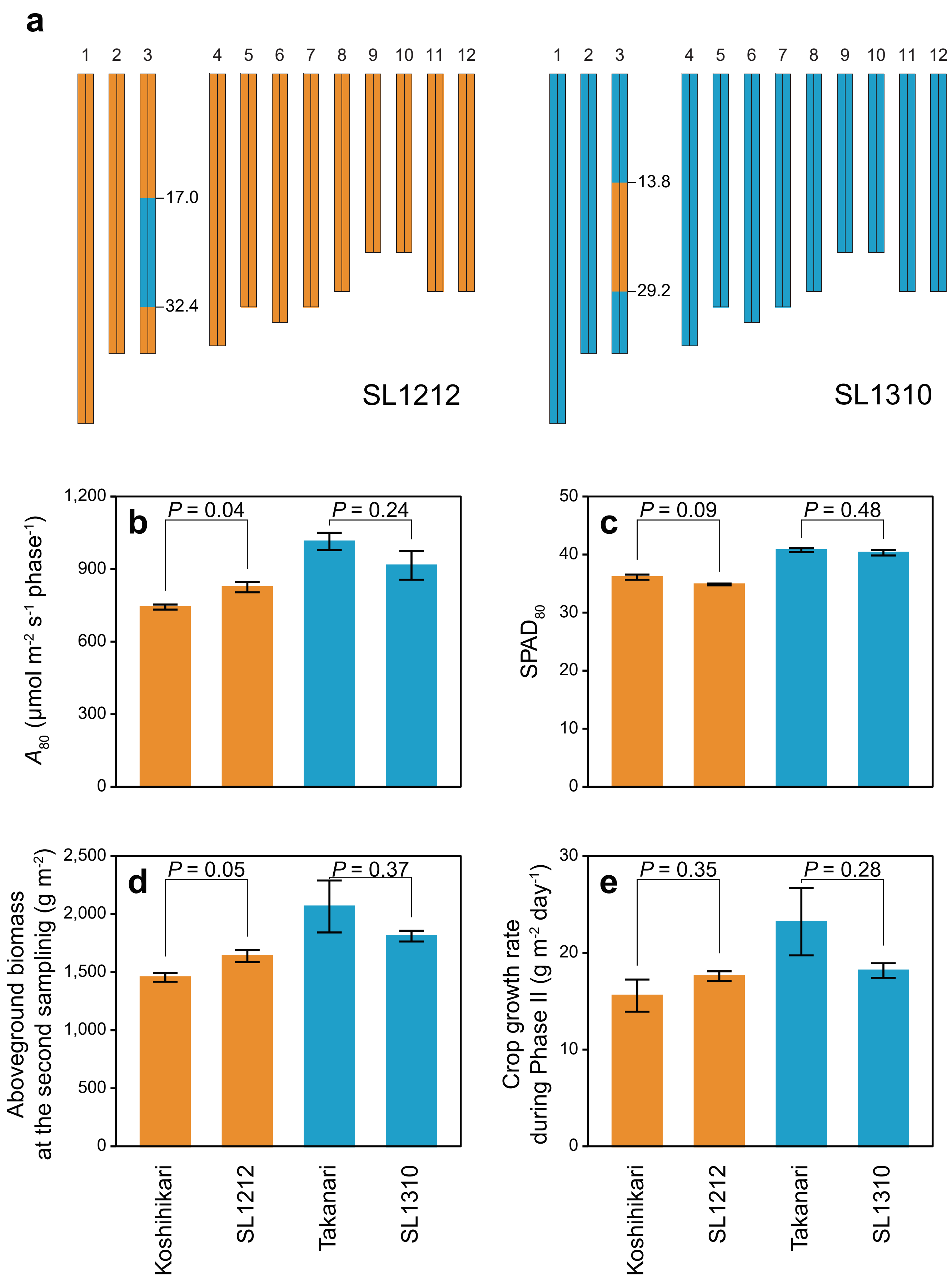

Supplementary Figure S6. Photosynthesis and bioimass accumulation properties of Koshihikari, SL1212, Takanari and SL1310. (**a**) Graphical genotypes of the CSSLs; orange, Koshihikari genomic region; blue, Takanari genomic region. The numerals represent physical positioins of the substituted genomic segments. The overlapping region between the lines was 12.2 Mb. (**b**) Accumulated *A* from 72 days after transplanting to D_{onset} (A_{80}). (**c**) Mean SPAD value before D_{onset} ($SPAD_{80}$). (**d**) Dry weight of aboveground biomass at the second sampling (AGB_{II}). (**e**) Crop growth rate during Phase II ($CGR\text{-}_{PhaseII}$). Error bars represent standard error (n = 3). Statistical differences were tested by Welch's two-sided *t*-test (n = 3).

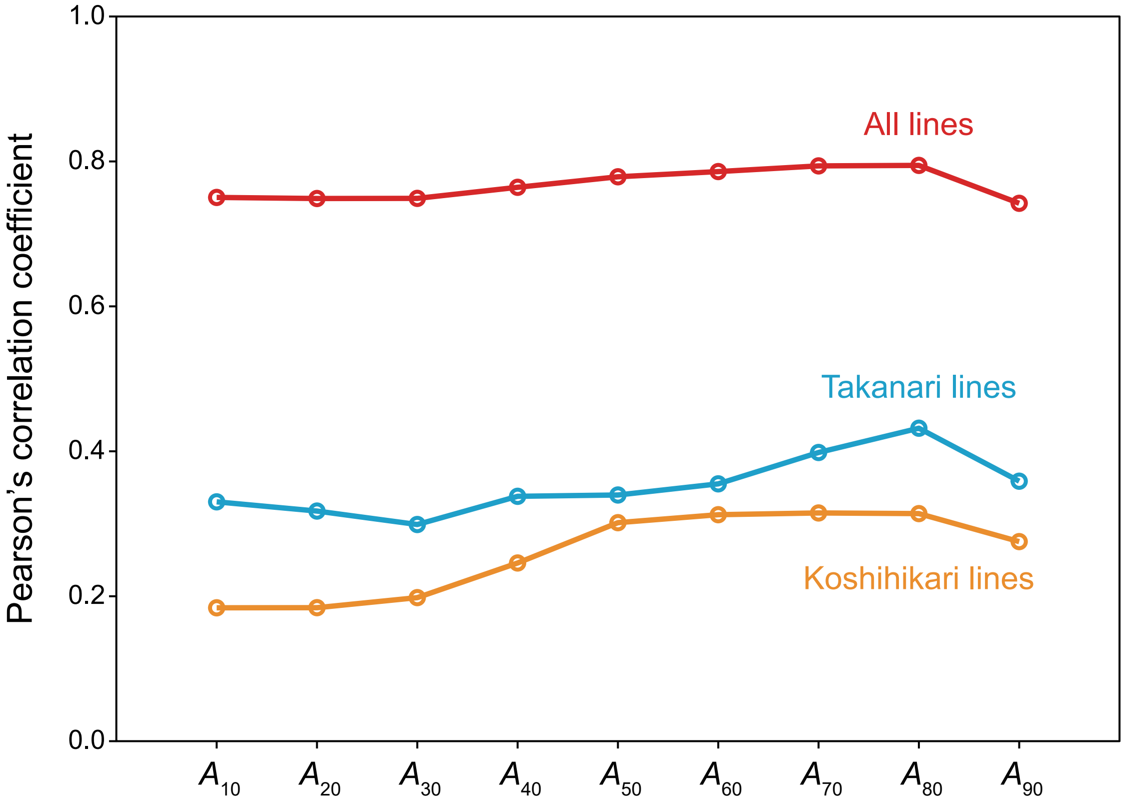

Supplementary Figure S7. Pearson's correlation coefficients of crop growth rate during Phase II ($CGR_{Phase\ II}$) with accumulated *A* after heading. Accumulated *A* was the integral from 72 days after transplanting (DAT) to D_{onset} (1 day before *A* declined below 10%-90% of maximum *A*). We selected A_{80} because it had the highst correlation in the combined data set and the Takanari line set and the second highest in the Koshihikari line set.

Supplementary Table S1. Standard deviations (SDs) of the traits shown in Figure 2.

Trait	Group	Phase or Sampling	SD
Integrated net CO_2 assmilation rate	Koshihikari lines	I	54.61
[μmol m^{-2} s^{-1} $phase^{-1}$]		II	79.83
	Takanari lines	I	65.98
		II	61.16
Mean single leaf area [cm^2]	Koshihikari lines	I	2.35
		II	4.24
	Takanari lines	I	3.49
		II	6.87
Aboveground biomass [g m^{-2}]	Koshihikari lines	1st	50.95
		2nd	141.86
	Takanari lines	1st	67.76
		2nd	144.70
Crop growth rate [g m^{-2} day^{-1}]	Koshihikari lines	I	0.72
		II	2.51
	Takanari lines	I	0.95
		II	2.17

Supplementary Table S2. Yield-related genes included in the suggestive genomic region of chromosome 3 (17.0-29.2 Mb). They were extracted from the gene list summarized by Wei et al. (2021)[1].

ID (RAP locus)	Gene	Start position [bp]	End position [bp]	Trait	Reference
Os03g0417700	*GL3.2/CYP78A5*	17,340,417	17,342,284	grain size	(2,3)
Os03g0418600	*Awn3-1*	17,410,000	17,412,951	awn length	(4)
Os03g0437200	*bsr-d1*	18,435,990	18,437,086	blast resistance	(5)
Os03g0576200	*qSE3/OsHAK21*	21,060,638	21,064,968	seed germination under salinity stress	(6)
Os03g0646900	*GL3.1/qGL3*	25,042,427	25,051,072	grain length	(7-9)
Os03g0700400	*LOX-3*	28,091,611	28,094,613	physiological trait	(10)
Os03g0706500	*OsTB1/SCM3*	28,428,504	28,430,438	lodging resistance	(11)
Os03g0707600	*SLR1/OsGAI/Slr1-d*	28,512,754	28,515,086	plant height	(12)

(1) Wei, X. et al. A quantitative genomics map of rice provides genetic insights and guides breeding. *Nat. Genet.* **53**, 243-253 (2021).
(2) Xu, F. et al. Variations in *CYP78A13* coding region influence grain size and yield in rice. *Plant Cell Environ.* **38**, 800-811 (2015).
(3) Zhao, K. et al. Genome-wide association mapping reveals a rich genetic architecture of complex traits in *Oryza sativa*. *Nat. Commun.* **2**, 467; 10.1038/ncomms1467 (2011).
(4) Li, B. et al. Fine Mapping of Two Additive Effect Genes for Awn Development in Rice (*Oryza sativa* L.). *PLoS One* **11**, e0160792; 10.1371/journal.pone.0160792 (2016).
(5) Li, W. et al. A natural allele of a transcription factor in rice confers broad-spectrum blast resistance. *Cell* **170**, 114-126 (2017).
(6) He, Y. et al. A quantitative trait locus, *qSE3*, promotes seed germination and seedling establishment under salinity stress in rice. *Plant J.* **97**, 1089-1104 (2019).
(7) Hu, Z. et al. A Kelch motif-containing serine/threonine protein phosphatase determines the large grain QTL trait in rice. *J. Integr. Plant Biol.* **54**, 979-990 (2012).
(8) Qi, P. et al. The novel quantitative trait locus *GL3.1* controls rice grain size and yield by regulating Cyclin-T1;3. *Cell Res.* **22**, 1666-1680 (2012).
(9) Zhang, X. et al. Rare allele of *OsPPKL1* associated with grain length causes extra-large grain and a significant yield increase in rice. *Proc. Natl. Acad. Sci. USA* **109**, 21534-21539 (2012).
(10) Shirasawa, K., Takeuchi, Y., Ebitani, T. & Suzuki, Y. Identification of gene for rice (*Oryza sativa*) seed lipoxygenase-3 involved in the generation of stale flavor and development of SNP markers for lipoxygenase-3 deficiency. *Breeding Sci.* **58**, 169-176 (2008).
(11) Yano, K. et al. Isolation of a novel lodging resistance QTL gene involved in strigolactone signaling and its pyramiding with a QTL gene involved in another mechanism. *Mol. Plant* **8**, 303-314 (2015).
(12) Huang, X. et al. Genomic architecture of heterosis for yield traits in rice. *Nature* **537**, 629-633 (2016).
